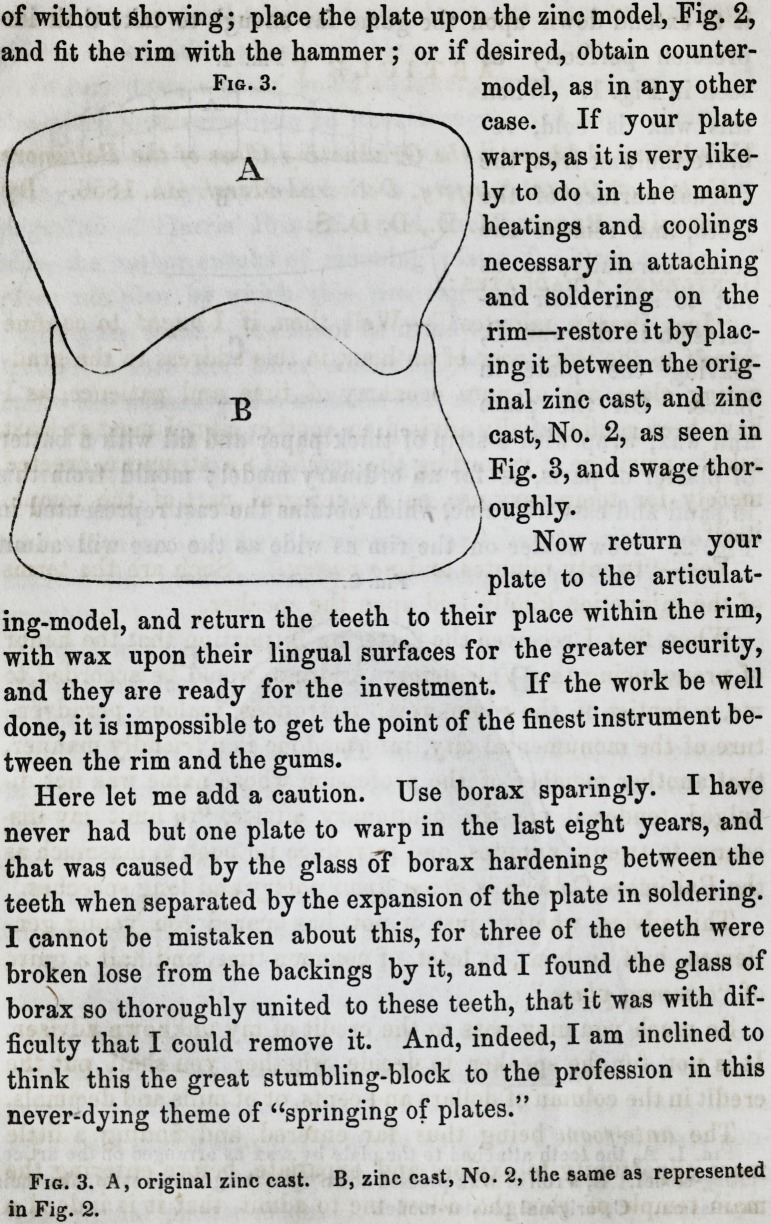# Manner of Swaging the Binding or Rim of Plates for Full Sets of Single Gum Teeth

**Published:** 1856-04

**Authors:** H. N. Case

**Affiliations:** dentist, Little Rock, Ark.


### ARTICLE IV.

*Manner of Swaging the Binding or Rim of Plates for full sets of Single Gum Teeth.* By H. N. Case, dentist, Little Rock, Ark.

Never having seen any given plan by which to *fit perfectly* the rim of suction plates for full sets of single gum teeth, and deeming it essential, if not absolutely indispensable to fine work, I send you the one by which I work, for publication, if you think it of sufficient interest to the profession.

Chapin A. Harris, in his "Principles and Practice," speaks, both of rimming and turning down plates, but I have never been able to make either plan available as given by him.

To turn down a plate would add very much to the finish of the piece, but very little to the strength, and then the plate cannot be fitted to the border with pliers after swaging, or changed in any way which it is often necessary to do. On page 725 of Harris' Principles and Practice, whence I got the idea, the author speaks of rimming plates for block teeth, but gives no plan by which this rim can be applied or fitted to single gum teeth. It cannot be fitted to the teeth themselves, neither before nor after soldering, without breaking them; hence the necessity of a metallic cast of the gums to fit the rim to. I make this rim to extend down upon the gums from $\frac{1}{8}$ to $\frac{3}{16}$ of an inch as the case will admit of.

The great superiority of this kind of work as regards cleanliness, strength, comfort in wearing, and beauty of finish, will suggest the great necessity of its being as perfectly done as possible.

But I will describe briefly. I strike up my plate in the usual manner, but lest this might be mistaken, I give it. First mould in fine sand from a plaster model, neatly trimmed and smoothly varnished, cast in this mould with zinc, and obtain counter-model, by immersing this to the proper depth, in melted lead and type-metal. Now cut your plate to extend as far up over the alveolar border as is desired, and swage thoroughly. When the swaging is complete, anneal and swage two or three times more, to prevent the plate from warping.

Now try the plate in the mouth, and complete the fitting with the pliers if necessary. Now take the articulating-model, select and fit the teeth nicely, put on backings, arrange the teeth upon the plate precisely as you wish them to stand when completed; and if the backings do not fit well to the plate, fill in with gold foil when you come to solder. Take the plate containing the teeth from the articulating-model, and place it upon the original plaster-model.

Now warm a roll of yellow wax, and place it around the plaster cast just above the plate, press it down upon the model, allowing it to extend down upon the gums far enough to take their impression perfectly as seen in Fig. 1. When this wax is cold, remove the wax from the lingual surface of the teeth, and remove the teeth carefully, so as not to spoil their impression in the wax B, leaving the plate in place. Oil the plate and wax, wrap with a strip of thick paper and fill with a batter of plaster of paris, as for an ordinary model; mould from this in sand and cast with zinc, which obtains the cast represented in Fig. 2. Now solder on the rim as wide as the case will admit

Fig. 1.

Fig. 2.

Fig. 1. A, the teeth attached to the plate by wax as arranged on the articulating-model. B, a roll of wax pressed down upon the gums so as to take their mpress on. C, original plaster-model.

Fig. 2. Representation of zinc cast, No. 2. A, perfect model of lingual surface of the plate. B B, perfect model of the gums to which the rim is to be nicely fitted.

of without showing; place the plate upon the zinc model, Fig. 2, and fit the rim with the hammer; or if desired, obtain counter-model, as in any other case. If your plate warps, as it is very likely to do in the many heatings and coolings necessary in attaching and soldering on the rim—restore it by placing it between the original zinc cast, and zinc cast, No. 2, as seen in Fig. 3, and swage thoroughly.

Fig. 3.

Now return your plate to the articulating-model, and return the teeth to their place within the rim, with wax upon their lingual surfaces for the greater security, and they are ready for the investment. If the work be well done, it is impossible to get the point of the finest instrument between the rim and the gums.

Here let me add a caution. Use borax sparingly. I have never had but one plate to warp in the last eight years, and that was caused by the glass of borax hardening between the teeth when separated by the expansion of the plate in soldering. I cannot be mistaken about this, for three of the teeth were broken lose from the backings by it, and I found the glass of borax so thoroughly united to these teeth, that it was with difficulty that I could remove it. And, indeed, I am inclined to think this the great stumbling-block to the profession in this never-dying theme of "springing of plates."

Fig. 3. A, original zinc cast. B, zinc cast, No. 2, the same as represented in Fig. 2.

## Figures and Tables

**Figure f1:**
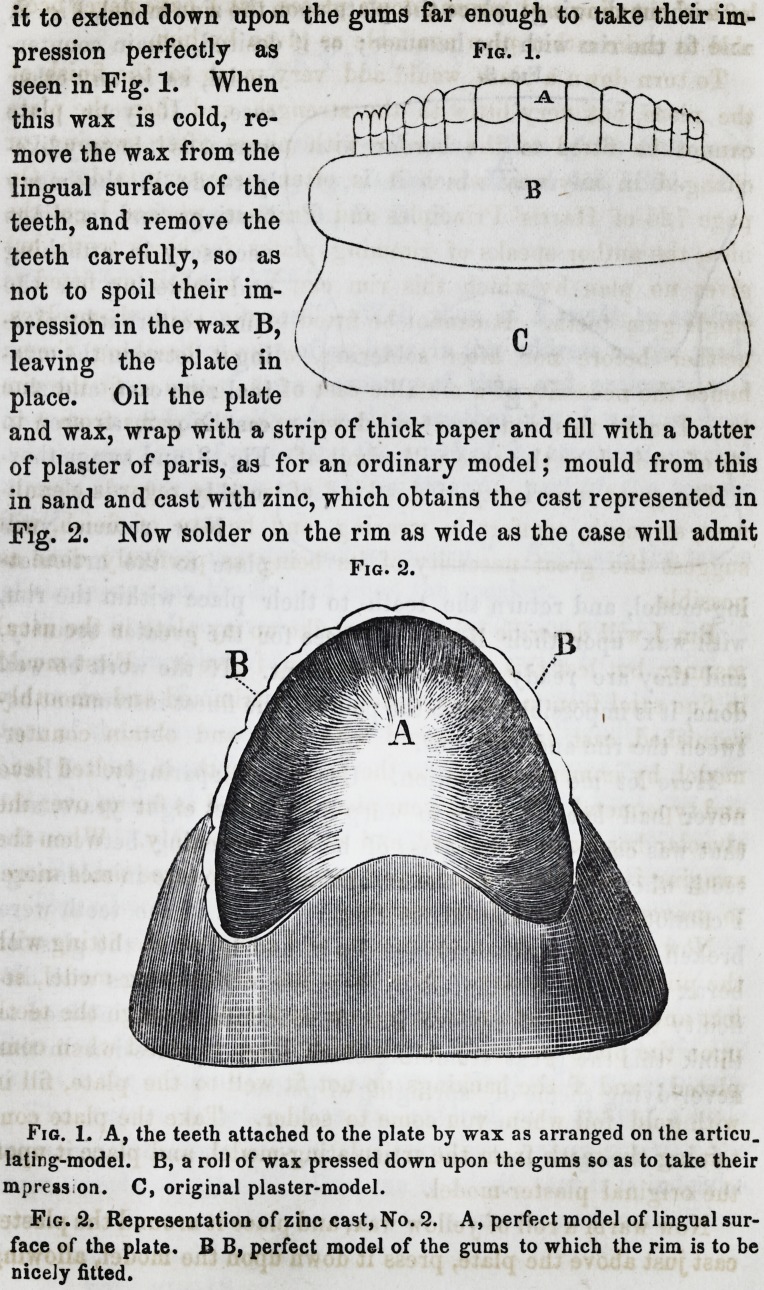


**Fig. 3. f2:**